# Nurses’ Knowledge and Attitudes Towards Addiction: A Descriptive Study

**DOI:** 10.1192/j.eurpsy.2026.10842

**Published:** 2026-07-31

**Authors:** I. Mosbah, H. ghabi, A. barketi, A. karmous, H. nefzi, R. kammoun, M. karoui, F. Ellouze

**Affiliations:** 1 Department of Psychiatry G, Razi hospital, Manouba; 2 El Manar University, Faculty of Medicine of Tunis; 3 Department of Psychiatry G, Razi hospital, Tunis, Tunisia

## Abstract

**Introduction:**

Addiction represents a major public health issue, often accompanied by stigma and misconceptions among healthcare providers. Nurses working in psychiatry, being at the frontline of patient care, play a crucial role in the management of psychiatric patients who are at more risk of substance use disorders.

**Objectives:**

This study aimed to assess the theoretical knowledge and attitudes of nurses regarding addiction and its impact on psychiatric care

**Methods:**

A cross-sectional descriptive study was conducted among 36 nurses working in adult and child psychiatry departments at Razi and Monji Slim hospitals. Items assessed theoretical knowledge of addiction (definition, causes, psychiatric impact) and attitudes towards patients with addictive disorders (reactions, ethical stance, communication style). Data were analyzed descriptively and expressed as frequencies and percentages.

**Results:**

The participants included 36 nurses, predominantly female (78%), mostly aged 20 to 40 years old, with 41% having less than 5 years of professional experience. obacco was the most common substance encountered (80.6%), followed by cannabis (72.2%), benzodiazepines (66.4%), and alcohol (50%). Buprenorphine, heroin, and cocaine were also reported, but with lower prevalence.

Regarding knowledge, 47% of nurses reported having good knowledge of addiction, 42% average, and only 11% very advanced. Addiction was mostly perceived as dependence (61.1%). 13.9% considered it a personal choice, while only a few viewed it as a chronic disease (11.1%). Social influence (91.7%) lack of prevention (58.3%) and psychiatric affections (52.8%) were considered the main causes, while genetic factors were less recognized (16.7%). All participants agreed that drug use worsens psychiatric disorders. Concerning attitudes, 61.1% reported remaining neutral and professional when facing withdrawal symptoms, while 19.4% avoided the patient and 2.8% expressed irritation. Trust-building strategies included clear communication (80.5%) and empathy (69.4%), although 5.6% admitted judging patients. Ethically, 44% responded with empathy without judgment, whereas 42% opted for strict advice.

**Conclusions:**

Nurses demonstrated partial knowledge of addiction, with limited understanding of its chronic bio-psycho-social nature and inconsistent attiude. These findings highlight the need for targeted training in addiction care to enhance knowledge, reduce stigma, and promote more supportive, patient-centered approaches in clinical practice.

**Disclosure of Interest:**

None Declared

